# Association between waist circumference change and incident chronic obstructive pulmonary disease among Chinese adults: a 10-year cohort study

**DOI:** 10.1038/s41598-022-23248-z

**Published:** 2022-11-01

**Authors:** Yun-Lei Ma, Han-Jun Zhao, Ying-Hao Su

**Affiliations:** 1 Department of Respiratory Medicine, Traditional Chinese Medicine Hospital of Hebei Province, 389 Zhongshan Road, Shijiazhuang, 050017 China; 2 Department of Respiratory Medicine, Fourth Hospital of Hebei Medical University, 12 Jiankang Road, Shijiazhuang, 050017 China

**Keywords:** Diseases, Respiratory tract diseases, Chronic obstructive pulmonary disease

## Abstract

The aim of our study was to investigate waist circumference (WC) change and the risk of incident chronic obstructive pulmonary disease (COPD) among Chinese adults. A total of 8164 participants aged > 18 years who attended health examinations with repeat measurements of WC and lung function [forced vital capacity (FVC), forced expiratory volume in 1 s (FEV_1_)] from 2010 to 2019 were recruited. WC change was categorized as ≤  − 2.5%, − 2.5 to 2.5%, 2.5% to 5% and > 5% according to sex. Modified Poisson regression models were used to assess the association of WC gain and the risk of COPD. During the 10-year follow-up, a total of 917 COPD cases were identified. From baseline to follow-up, the mean FEV_1_ decreased from 3.20 to 2.79L among male participants and 2.28–1.95L among female participants. Compared with participants who did not have abdominal obesity, at either, baseline or follow-up, participants with abdominal obesity of both sexes after the follow-up were associated with a greater risk of COPD regardless of abdominal obesity at baseline. The risk of incident COPD increased 19% among male participants (*RR* = 1.19, 95%*CI* = 1.04–1.48) and 14% among female participants (*RR* = 1.14, 95%*CI* = 1.01–1.40) when WC gain increased > 5% during the 10-year follow-up. The COPD risk decreased 18% among male participants with a WC change ≤  − 2.5% (*RR* = 0.82, 95%*CI* = 0.67–0.99). The risk of incident COPD was positively associated with increasing WC among Chinese adults of both sexes.

## Introduction

The morbidity and mortality of COPD have increased dramatically worldwide in recent decades^1^. The World Health Organization (WHO) predicted that COPD would go from being the fourth leading cause of death in 2004 to the third by 2030, with more than 90% of the morbidity and mortality of COPD occurring in low- and middle-income countries^2,3^. As shown in a recent meta-analysis on Chinese individuals, the age-adjusted prevalence of COPD defined by spirometry tests increased rapidly from 8.2% in 2002–2004 to 13.7% in 2012–2015^4^.

Available studies have suggested that several factors are associated with an increased risk of COPD, including higher BMI (body mass index), increasing age, tobacco smoking, household air pollution, and occupational exposures^5–9^. The association of general obesity (BMI ≥ 30 kg/m^2^) and the risk of COPD have received much attention for a long time, but there are no consistent conclusions at present. On the one hand, some studies suggested that BMI was inversely associated with the risk of COPD^9–11^. On the other hand, the “obesity paradox” of COPD in many studies showed that overweight/obesity was helpful to improve COPD patient survival^12,13^. However, the common definition for obesity or adiposity relies on BMI, which is simple and reasonable. WC, as another measurement of obesity, better reflects the distribution of fat tissue throughout the body. However, there is a lack of studies regarding the association of WC with lung function, and available studies only conducted among children and adolescents showed complex results. Marga et al.^14^ found that a large WC was not associated with FEV_1_ (forced expiratory volume in 1 s) or FVC (forced vital capacity) among children aged 8 years, while Feng et al.^15^ suggested that WC was inversely associated with lung function in Chinese children and adolescents.

Thus, our study aimed to assess the association of lung function and the risk of incident COPD with 10-year changes in adiposity as measured by WC among Chinese adults. Furthermore, this relationship was investigated by sex.

## Materials

### Study population

This longitudinal cohort consisted of 11,357 participants residing in Shijiazhuang city which is the provincial capital city of Hebei Province in China. All participants attended health examinations conducted in 21 public medical institutions during 2010–2019. The survey was initially conducted from March to April 2010 to March to April 2011 for participants aged over 18 years. However, participants with missing data, including information on age, WC, FEV_1_ or FVC (*n* = 2589), as well as pregnant and breastfeeding participants (*n* = 157) were excluded. Additionally, participants were excluded if they had underlying lung disease asthma or asthma-like symptoms, including wheezing, nocturnal chest tightness, attacks of breathlessness after activity, at rest or at night, or the use of inhaled or oral medicine for breathing problems (*n* = 392). Considering possible confounding by disease and metabolic WC change, we also excluded participants who had diabetes (*n* = 52) and cancer (*n* = 3). Finally, our study included 8164 participants (3929 men and 4235 women) to estimate the risk of incident COPD with changes in WC during the 10-year follow-up.

Ethical approval was obtained from the ethics committees of all participating institutions and all participants provided written informed consent. This study was conducted according to the guidelines laid down in the Declaration of Helsinki and all procedures involving human subjects were approved by the Ethics Committee of the Traditional Chinese Medicine Hospital of Hebei Province.

### Data collection

Before the health examination, all participants needed to complete an overnight fast. Questionnaires were used to collect baseline information on social and demographic factors, including age, sex, educational level, smoking habits, alcohol consumption and physical activity. The same questionnaires were employed for the information collection in each year. Educational level was categorized as low level (below senior high school) and high level (college or university degree and master’s degree or above). Smoking status was categorized as current smoking, ever smoking or never smoking. Alcohol consumption was categorized as drinker (alcohol consumption 12 or more times in the last year) and non-drinker (alcohol consumption fewer than 12 times in the last year). Area of residence was categorized as urban and rural living areas. Metabolic equivalents (METs) were calculated by codes and the detailed questions of the physical activity survey have been published elsewhere^16–18^. The same measurements of the health examination at baseline were taken after 10 years of follow-up.

All measurements of WC and BMI were collected according to the World Health Organization standard^19,20^. WC was measured with gentle breathing at the midpoint between the lowest rib and the iliac crest to the nearest 0.1 cm. The difference value (D-value) in WC was calculated by subtracting the WC measured at baseline from that measured at follow-up. The percent of WC gain > 0% indicated that WC increased from baseline to follow-up; the greater the percentage of WC gain was, the greater the increase in WC. Weight and height were measured with participants in light clothing and without shoes. All subjects were measured by investigators who received standardized measurement training using the same standard height-weight scale (Shengyuan SY-L30, China). The measurements of weight and height were corrected to the nearest 0.5 kg and 0.1 cm, respectively. Body mass index (BMI) was calculated as the ratio of weight (kg) to height squared (m^2^).

Overnight fasting blood was collected into vacuum tubes to assess fasting plasma glucose (FPG), total cholesterol (TC), triglycerides (TGs) and high-density lipoprotein cholesterol (HDL-C). The storage and measurement methods were in accordance with clinical practices standards^21^.

Lung function was measured by a respiratory physician using the same spirometer (MasterScreen Pneumo, Jaeger, Germany) and all examinations were in accordance with American Thoracic Society recommendations^22^. First, all participants were guided in practicing the exhalations before the examination of lung function. Then, pre- and postbronchodilator forced expiratory volume in one second (FEV_1_) and forced vital capacity (FVC) were measured as the primary outcomes and the ratio of these two measurements (FEV_1_: FVC) was calculated for each subject to assess the risk of COPD. Participants were asked to return on another day for an additional spirometry test if the result of the examination was regarded as low quality. According to the study design, we regarded the spirometry examination values out of the medical reference range (< 2.5^th^ percentiles and > 97.5^th^ percentiles) as low quality.

### Outcome definitions

Abdominal obesity for Chinese adults was defined by a WC ≥ 90 cm for men and WC ≥ 80 cm for women according to the criteria of the Working Group on Obesity in China^23^.

According to the Global Initiative for Chronic Obstructive Lung Disease (GOLD) criteria, COPD was defined as a postbronchodilator FEV_1_: FVC < 70%^24^. Combined with the diagnostic criteria for COPD in China, we also considered patients’ history of illness and the results of X-ray and biochemical examinations when participants were diagnosed with COPD^25^. Patients with COPD were defined as participants who reported a previous diagnosis of COPD through spirometry testing by a physician. Participants were also defined as COPD patients by self-reporting that they received any inhaled short-acting or long-acting bronchodilator or corticosteroid therapy before.

### Statistical analysis

All continuous variables are presented as the means (standard deviations) and categorical data are presented as numbers (percentages). The Kruskal–Wallis test or chi-square test was used to estimate the differences in variables in each group at baseline. To examine the association of dynamic WC change and incident COPD risk in detail, the range of the percentage of WC change was divided into small categories: ≤  − 2.5%, − 2.5% to 2.5%, 2.5% to 5% and > 5%.

Three Poisson regression models were used to assess the association of the risk of COPD and WC gain by relative risks (*RRs*) and 95% confidence intervals (*CIs*)^26,27^. For the first part of the analysis, a WC gain of − 2.5% to 2.5% was regarded as the reference group. Participants with a normal WC at both baseline and follow-up were regarded as the reference group in the second part of the analysis. All potential confounding factors in the regression models were adjusted for based on the baseline variables. Model 1 adjusted for age first. Then, Model 2 adjusted for age as well as education level, smoking status, alcohol consumption, and physical activity. Finally, Model 3 adjusted for all variables in Model 2 as well as body mass index, systolic blood pressure (SBP), diastolic blood pressure (DBP), fasting plasma glucose (FPG), total cholesterol (TC), triglycerides (TGs), high density lipoprotein cholesterol (HDL-C), forced expiratory volume in 1 s (FEV_1_) and forced vital capacity (FVC).

All analyses were performed by Stata, version 12.0 (Stata Corporation, College Station, TX, USA). A two-tailed *p* value less than 0.05 was regarded as significant.

## Results

A total of 8164 participants were included in this study. The baseline characteristics of the study population by WC gain category are shown in Table 1. The mean (SD) age for men and women was 37.6 (8.12) years and 37.3 (8.01) years, respectively, and the mean (SD) WC at baseline was 82.3 (9.56) cm and 72.4 (9.44) cm, respectively. During a median follow-up of 8.4 years, a total of 917 COPD cases (516 men) were identified.

**Table 1:** Baseline characteristics of study participants stratified by percentage of waist circumference (WC) change from baseline to follow-up.

Baseline	Percent of WC gain (%)				*p*-value
	≤ − 2.5	− 2.5 to 2.5	2.5 to 5	> 5	
Men (*n* = 3929)	782	1190	570	1387	
Age (years)	37.7 (9.02)	37.9 (8.62)	37.2 (8.34)	37.4 (8.71)	0.142
High educational (%)	128 (16.4)	226 (19.0)	97 (17.1)	221 (15.9)	0.201
Smoking (%)	558 (71.4)	852 (71.6)	412 (72.3)	986 (71.1)	0.961
Alcohol drinking (%)	238 (30.4)	361 (30.3)	178 (31.2)	456 (32.9)	0.498
Physical activity (MET/hours per week)	285 (25.8)	278 (27.9)	257 (28.9)	255 (29.7)	< 0.001
Urbanization (%)	267 (34.2)	410 (34.5)	195 (34.2)	485 (35.0)	0.978
BMI (kg/m^2^)	23.0 (3.36)	23.2 (3.24)	23.4 (3.11)	23.6 (2.88)	0.067
WC (cm)	85.1 (10.5)	83.9 (9.80)	82.8 (9.44)	81.9 (8.65)	0.044
SBP (mm Hg)	118.8 (11.8)	118.3 (11.7)	118.1 (10.4)	117.2 (10.5)	0.105
DBP (mm Hg)	76.2 (7.68)	74.8 (7.80)	74.4 (7.32)	73.7 (7.67)	0.041
FPG (mg/dl)	89.5 (14.4)	91.2 (20.1)	90.4 (17.8)	92.3 (18.2)	0.079
TC (mmol/L)	4.38 (0.87)	4.38 (0.91)	4.36 (0.88)	4.22 (0.91)	0.008
TG (mmol/L)	1.77 (1.32)	1.63 (1.12)	1.64 (1.18)	1.68 (0.89)	0.101
HDL-C (mmol/L)	1.16 (0.26)	1.10 (0.28)	1.11 (0.25)	1.13 (0.27)	0.112
FEV_1_ (L)	3.21 (0.50)	3.23 (0.54)	3.20 (0.61)	3.19 (0.70)	0.282
FVC (L)	3.13 (0.43)	3.16 (0.41)	3.11 (0.54)	3.18 (0.37)	0.173
FEV_1_/FVC (%)	93.4	92.2	90.6	91.3	
Women (*n* = 4235)	860	1058	567	1750	
Age (years)	37.3 (8.23)	37.3 (8.45)	37.8 (8.61)	37.0 (8.98)	0.134
High educational (%)	182 (21.2)	198 (18.7)	113 (19.9)	301 (17.2)	0.087
Smoking (%)	3 (0.35)	2 (0.19)	1 (0.18)	2 (0.11)	0.640
Alcohol drinking (%)	4 (0.47)	6 (0.57)	4 (0.71)	4 (0.23)	0.364
Physical activity (MET/hours per week)	268 (24.8)	265 (28.7)	243 (25.6)	246 (26.4)	< 0.001
Urbanization (%)	299 (34.8)	364 (34.4)	200 (35.2)	600 (34.3)	0.975
BMI (kg/m^2^)	22.5 (3.54)	22.3 (3.32)	22.2 (3.31)	22.6 (3.32)	0.328
WC (cm)	74.2 (9.67)	71.8 (9.56)	70.4 (8.90)	68.3 (8.89)	< 0.001
SBP (mm Hg)	116.5 (11.3)	115.7 (11.7)	114.3 (11.6)	115.8 (11.5)	0.099
DBP (mm Hg)	74.2 (7.45)	74.2 (7.55)	73.7 (7.69)	74.1 (7.32)	0.110
FPG (mg/dl)	89.8 (14.7)	90.8 (17.6)	91.4 (18.6)	91.1 (18.0)	0.073
TC (mmol/L)	4.44 (0.99)	4.49 (0.97)	4.44 (0.97)	4.41 (0.87)	0.093
TG (mmol/L)	1.65 (1.13)	1.69 (1.12)	1.67 (1.06)	1.68 (0.98)	0.236
HDL-C (mmol/L)	1.21 (0.27)	1.19 (0.27)	1.23 (0.29)	1.22 (0.26)	0.082
FEV_1_ (L)	2.30 (0.44)	2.29 (0.48)	2.26 (0.52)	2.28 (0.61)	0.249
FVC (L)	2.51 (0.47)	2.56 (0.45)	2.59 (0.51)	2.55 (0.48)	0.190
FEV_1_/FVC (%)	90.4	91.6	90.6	91.9	

*BMI* body mass index, *WC* waist circumference, *SBP* systolic blood pressure, *DBP* diastolic blood pressure, *FPG* fasting plasma glucose, *TC* total cholesterol, *TG* triglyceride, *HDL-C* high-density lipoprotein cholesterol, *FEV*_*1*_ forced expiratory volume in one second, *FVC* forced vital capacity, *FEV*_*1*_*/FVC* the ratio of forced expiratory volume in one second and forced vital capacity.

Our study investigated COPD risk according to dynamic WC gain. As shown in Tables 2 and 3, the RRs for COPD increased significantly with increasing percentage of WC gain among both male and female participants (*p* for trend < 0.01). Compared with the group of − 2.5% to 2.5% WC change, the adjusted RR (95% CIs) for COPD with a WC gain > 5% was 1.19 (95%*CI* = 1.04–1.48) among men and 1.14 (95%*CI* = 1.01–1.40) among women. The COPD risk significantly decreased among men with a WC loss over 2.5% (*RR* = 0.82, 95%*CI* = 0.67–0.99).

**Table 2:** Risk of chronic obstructive pulmonary disease (COPD) by percentage of waist circumference (WC) change for men.

Percent of WC gain (%)	Total		COPD cases	Incidence (%)	RR (95% CI)		
					Model 1^a^	Model 2^b^	Model 3^c^
**Total**							
≤ − 2.5	782		85	10.9	0.88 (0.62, 1.04)	0.85 (0.60, 0.99)	0.82 (0.67, 0.99)
− 2.5 to 2.5	1190		152	12.8	Reference	Reference	Reference
2.5 to 5	570		75	13.1	1.07 (0.89, 1.30)	1.01 (0.79, 1.21)	0.96 (0.81, 1.21)
** > **5	1387		204	14.7	1.11 (0.91, 1.35)	1.13 (0.93, 1.39)	1.19 (1.04, 1.48)
P for trend					< 0.001	< 0.001	< 0.001
**Non-abdominal obesity at baseline**							
≤ − 2.5	511		43	8.4	0.89 (0.64, 1.02)	0.83 (0.55, 0.96)	0.83 (0.60, 1.01)
− 2.5 to 2.5	885		94	10.6	Reference	Reference	Reference
2.5 to 5	451		51	11.3	1.15 (0.87, 1.39)	1.12 (0.77, 1.29)	1.09 (0.75, 1.22)
> 5	1252		159	12.7	1.19 (1.02, 1.36)	1.15 (0.98, 1.35)	1.15 (1.06, 1.38)
P for trend					< 0.001	< 0.001	< 0.001
**Abdominal obesity at baseline**							
≤ − 2.5	271		42	15.4	0.89 (0.64, 1.13)	0.80 (0.59, 1.10)	0.86 (0.65, 1.14)
− 2.5 to 2.5	305		58	19.0	Reference	Reference	Reference
2.5 to 5	119		24	20.2	1.05 (0.75, 1.48)	1.04 (0.73, 1.50)	1.13 (0.80, 1.61)
** > **5	135		45	33.3	1.45 (1.07, 1.94)	1.43 (1.03, 1.96)	1.55 (1.14, 2.12)
P for trend					< 0.001	< 0.001	< 0.001

Data are relative risk (RR) and 95%CIs.

^a^Adjusted for age at baseline.

^b^Adjusted for variables in model 1 as well as education level, smoking status, alcohol drinking, and physical activity at baseline.

^c^Adjusted for variables in model 2 as well as body mass index, systolic blood pressure, diastolic blood pressure, fasting plasma glucose, total cholesterol, triglycerides, high density lipoprotein cholesterol, forced expiratory volume in one second and forced vital capacity at baseline.

**Table 3:** Risk of chronic obstructive pulmonary disease (COPD) by percentage of waist circumference (WC) change for women.

Percent of WC change (%)	Total	COPD cases	Incidence (%)	RR (95% CI)		
				Model 1^a^	Model 2 ^b^	Model 3 ^c^
**Total**						
≤ ** − **2.5	860	74	8.6	0.88 (0.75, 1.02)	0.98 (0.81, 1.19)	0.98 (0.83, 1.19)
** − **2.5 to 2.5	1058	110	10.4	Reference	Reference	Reference
2.5 to 5	567	44	7.8	0.94 (0.79, 1.11)	1.03 (0.84, 1.28)	1.06 (0.85, 1.30)
> 5	1750	173	9.9	1.07 (0.95, 1.20)	1.12 (0.96, 1.31)	1.14 (1.01, 1.40)
P for trend				0.005	0.007	< 0.001
**Non-abdominal obesity at baseline**						
≤ ** − **2.5	292	10	3.4	0.87 (0.66, 1.17)	0.90 (0.62, 1.20)	0.94 (0.65, 1.34)
** − **2.5 to 2.5	471	22	4.7	Reference	Reference	Reference
2.5 to 5	277	5	1.8	0.78 (0.56, 1.08)	0.83 (0.56, 1.23)	0.92 (0.61, 1.35)
> 5	1045	49	4.7	1.02 (0.81, 1.29)	1.04 (0.79, 1.38)	1.03 (0.76, 1.39)
P for trend				< 0.001	0.008	< 0.001
**Abdominal obesity at baseline**						
≤ ** − **2.5	568	64	11.3	0.82 (0.69, 0.98)	0.95 (0.77, 1.18)	0.95 (0.76, 1.20)
** − **2.5 to 2.5	587	88	14.9	Reference	Reference	Reference
2.5 to 5	290	39	13.4	1.02 (0.83, 1.30)	1.08 (0.83, 1.42)	1.10 (0.82, 1.43)
> 5	705	124	17.6	1.28 (1.05, 1.56)	1.24 (1.02, 1.52)	1.27 (1.01, 1.35)
P for trend				0.009	0.004	< 0.001

Data are relative risk (RR) and 95%CIs.

^a^Adjusted for age at baseline.

^b^Adjusted for variables in model 1 as well as education level, smoking status, alcohol drinking, and physical activity at baseline.

^c^Adjusted for variables in model 2 as well as body mass index, systolic blood pressure, diastolic blood pressure, fasting plasma glucose, total cholesterol, triglycerides, high density lipoprotein cholesterol, forced expiratory volume in one second and forced vital capacity at baseline.

Moreover, participants were analyzed according to whether they had abdominal obesity at baseline. As shown in Tables 2 and 3, the risk of COPD increased 55% (*RR* = 1.55, 95%*CI* = 1.14–2.12) and 27% (*RR* = 1.27, 95%*CI* = 1.01–1.35) compared to the group of -2.5% to 2.5% WC gain among male and female participants with abdominal obesity at baseline, respectively.

**Figure 1:**
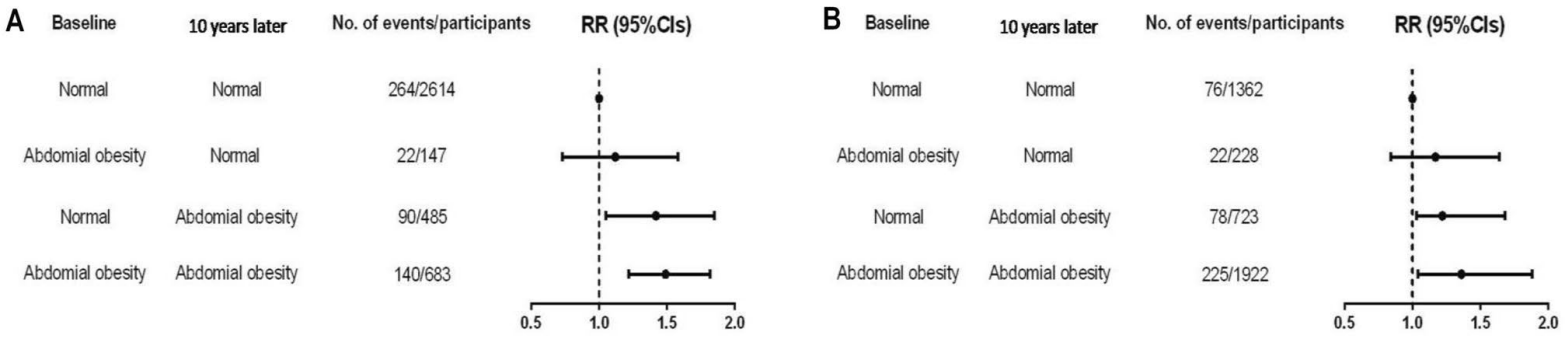
Risk of chronic obstructive pulmonary disease (COPD) by abdominal obesity among male (**A**) and female (**B**) participants at baseline and follow-up.

The risk of incident COPD by changes in abdominal obesity status was also investigated in our study. As shown in Fig. 1, the risk of incident COPD was significantly greater for both male and female participants who had abdominal obesity at the follow-up regardless of their abdominal obesity status at baseline than for participants who did not have abdominal obesity at either baseline or follow-up. The risk of incident COPD increased 42% (*RR* = 1.42, 95%*CI* = 1.05–1.85) for men (Fig. 1A) and 22% (*RR* = 1.22, 95%*CI* = 1.03–1.68) for women (Fig. 1B) with a normal WC at baseline and abdominal obesity at the follow-up. Additionally, the risk of new-onset COPD was more significant among male and female participants with abdominal obesity at both baseline and follow-up.

**Figure 2:**
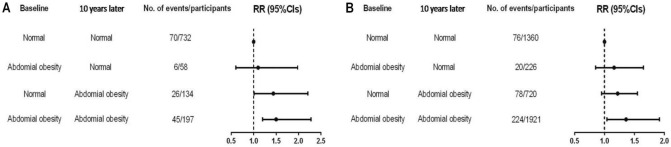
Sensitivity analysis of association between change of waist circumference (WC) and the risk of chronic obstructive pulmonary disease (COPD) among male (**A**) and female (**B**) participants who reported never smoking.

Finally, our study re-estimated the risk of incident COPD with a change in abdominal obesity status to exclude the effect of cigarette smoking on incident COPD. As shown in Fig. 2, the risk of COPD onset was basically consistent with the results for men (*RR* = 1.44, 95%*CI* = 1.01–2.21) (Fig. 2A) and women (*RR* = 1.36, 95%*CI* = 1.04–1.92) (Fig. 2B). The similar risk suggested an independent effect of WC on incident COPD.

## Discussion

To our knowledge, this is the first long-term cohort study to investigate the association of dynamic changes in WC and the incidence of COPD among Chinese adults. This study showed that WC gain was significantly and positively associated with the risk of COPD in Chinese adults. Additionally, our study suggested a dose–response relationship between WC gain and incident COPD for both male and female participants regardless of abdominal obesity status at baseline after adjusting for related covariates at baseline.

With the implementation of the opening and reformation policies in 1978, Chinese economy entered a rapid period of development^28^. According to the reports of the National Bureau of Statistics, gross domestic product per capita increased from 381 Yuan in 1978 to 38,420 Yuan in 2012^29^. The patterns in changes in dietary and physical activity have led to a higher prevalence of obesity in China during past decades^30^. Available evidence showed that the age-adjusted prevalence of abdominal obesity increased from 9.60 to 35.3% for men and from 30.5 to 51.7% for women during the 1993–2011 China Health and Nutrition Survey^31^. Furthermore, the age-adjusted prevalence of COPD defined by spirometry tests in China increased rapidly from 8.2% in 2002–2004 to 13.7% in 2012–2015^4^. Unfortunately, the results of our study suggested that abdominal obesity was closely associated with low levels of FEV_1_ and FVC and a high risk of incident COPD. Hence, comprehensive strategies aimed at the prevention of abdominal obesity are urgently needed to reduce the increase in the societal burden of lung diseases.

Few studies have assessed the relationship between abdominal obesity and lung function and related diseases^14,15,32^. Pan et al. found that central adiposity and WC, but not general adiposity and BMI, were independently associated with lower pulmonary function and a higher risk of restrictive respiratory defects in older Chinese individuals^32^. The detailed data showed that participants with only central adiposity had lower pulmonary function than those with neither central nor general obesity (adjusted βs ranged from − 2.85 to − 6.02 for FEV_1_% and FVC%, adjusted ORs ranged from 1.14 to 1.70, all *P* < 0.05). Another study conducted among Chinese children showed that waist-to-chest ratio (WCR) and WC was inversely associated with lung function. The results showed that each 0.01 increase in WCR was associated with an 8.14 ml decrease in FVC, a 9.36 ml decrease in FEV_1_, and a 6.54% decrease in FEV_1_/FVC^15^. These results were consistent with those of our study showing that a WC increase was positively associated with low lung function and the incidence of COPD. In contrast, Marga et al. found that a large WC was not associated with FEV_1_ and FVC among 8-year-old children, but they suggested that this association may change over the course of life from childhood to adulthood^14^. However, all of these studies focused only on the association of WC and lung function at a cross-sectional level, and there was a lack of estimation of the relationship of dynamic changes in WC with lung function and related diseases. Our study first used the percentage of WC gain to investigate lung function and the risk of incident COPD in a Chinese population and found that both men and women with increased WC gain during the 10-year follow-up shared a significantly increased risk of COPD after adjustment for important covariates.

Two potential mechanisms may explain the associations of WC gain with the increased risk of COPD. On the one hand, WC gain can affect lung function through mechanical effects on the lungs. Adiposity accumulation in the abdominal and thoracic regions may directly reduce vital capacity by limiting the room for lung expansion during inspiration and ultimately lead to expiratory flow limitation^33^. These mechanisms are also likely to explain the different risks of COPD between sexes, which were consistent with previous studies^34,35^, because men tend to accumulate more fat mass in the abdominal area than women^36^. On the other hand, WC gain can damage lung tissue by inflammatory processes, and fat tissue is a source of inflammatory mediators that can impair lung function and decrease airway diameter^37,38^. The major cause of COPD is the drop in FEV_1_, which is closely associated with lung inflammation related to high serum levels of C-reactive protein (CRP)^39^. Abdominal obesity is the main factor that increases CRP concentrations resulting from adiposity fat accumulation^40^. Systemic inflammation might lead to reduced lung function given that CRP and interleukin-6 (IL-6) are expressed in inflammatory lung epithelial cells^41^.

### Strengths and limitations

Our study has several strengths, including the large sample size, long-term follow-up and prospective study design. The included participants were all free of COPD and other lung diseases at baseline, which benefited the true investigation of the relationship between changes in WC and the risk of incident COPD.

However, there were also some limitations in this study. First, the study participants were recruited from one city in northern China, which may be due to a lack of the representation of Chinese adults. However, more than 91% of the Chinese population is of Han ethnicity, and participants in our study mainly included individuals of Han ethnicity (more than 93%). Hence, lifestyle and dietary factors were similar between individuals. In addition, smoking, drinking and physical activity levels were self-reported, which may have misestimated the rates due to the use of retrospective questionnaires. Finally, although our study adjusted for related covariates, other potential confounders were not adjusted for.

## Conclusions

In conclusion, this study provides epidemiological evidence to better understand the effect of changes in WC on the risk of incident COPD. Changes in WC are a significant predictor of incident COPD in Chinese adults. Maintaining a healthy lifestyle and regular physical activity are two important and common ways to reduce the risk of WC gain.

## Data Availability

The datasets used and/or analyzed during the current study available from the corresponding author on reasonable request.
